# Physiological Responses of Salinized Fenugreek (*Trigonella* *foenum-graecum* L.) Plants to Foliar Application of Salicylic Acid

**DOI:** 10.3390/plants10040657

**Published:** 2021-03-30

**Authors:** Reda E. Abdelhameed, Arafat Abdel Hamed Abdel Latef, Rania S. Shehata

**Affiliations:** 1 Botany and Microbiology Department, Faculty of Science, Zagazig University, Zagazig 44519, Egypt; rshahta@jazanu.edu.sa; 2 Department of Biology, Turabah University College, Turabah Branch, Taif University, P.O. Box 11099, Taif 21944, Saudi Arabia; 3 Biology Department, Faculty of Science, Jazan University, Jizan 45142, Saudi Arabia

**Keywords:** antioxidant enzymes, phenolics, fenugreek, flavonoids, proline, salicylic acid, salt stress, shikimic acid

## Abstract

Considering the detrimental effects of salt stress on the physiological mechanisms of plants in terms of growth, development and productivity, intensive efforts are underway to improve plant tolerance to salinity. Hence, an experiment was conducted to assess the impact of the foliar application of salicylic acid (SA; 0.5 mM) on the physiological traits of fenugreek (*Trigonella* *foenum-graecum* L.) plants grown under three salt concentrations (0, 75, and 150 mM NaCl). An increase in salt concentration generated a decrease in the chlorophyll content index (CCI); however, the foliar application of SA boosted the CCI. The malondialdehyde content increased in salt-stressed fenugreek plants, while a reduction in content was observed with SA. Likewise, SA application induced an accumulation of proline, total phenolics, and flavonoids. Moreover, further increases in total free amino acids and shikimic acid were observed with the foliar application of SA, in either control or salt-treated plants. Similar results were obtained for ascorbate peroxidase, peroxidase, polyphenol oxidase, and catalase with SA application. Hence, we concluded that the foliar application of SA ameliorates salinity, and it is a growth regulator that improves the tolerance of fenugreek plants under salt stress.

## 1. Introduction

Abiotic stresses have been documented as the chief threat to agricultural efficiency all over the world. Anthropogenic activities in the developmental era have intensified the degradation of the agricultural system and its productivity due to the foremost abiotic stresses, such as extreme temperatures, salinity, drought, nutrients (deficiency and excess), metals/metalloids, and UV radiation [1,2,3,4], which can potentially influence almost all plant metabolic processes and which may negatively affect 70% of crops’ yields [5]. One of these abiotic stresses is salinity, which threatens the agricultural system and affects all physiological processes, from seed germination to plant development, leading to reduced growth and yield [6,7,8]. The detrimental effects of salinity on plant physiology are associated with specific low water potential in soil solution (drought stress), ion effects, nutritional disparities, forwarding of energy from growth to pull out pure water from the saline water and to create defensive chemicals, or a combination of these altered factors [9,10]. 

Sensitivity to salt stress is mainly due to oxidative stress, which arises from a disturbance of the balance between the production and elimination of reactive oxygen species (ROS) such as superoxide, hydrogen peroxide, and hydroxyl radicals [11]. Furthermore, lipids, specifically polyunsaturated fatty acids, are sensitive to deterioration by ROS, inducing changes in the structural and functional properties of the cells [12,13]. Previous studies have confirmed the accumulation of lipid peroxidation products in salt-stressed plants, and the leakiness of membranes [14,15]. 

Recently, great efforts have been exerted to increase the protective mechanisms of plants against salt stress. One of these promising efforts is the application of phytohormones, such as salicylic acid (SA), which is a phenolic phytohormone that acts as a signaling molecule [16]. SA plays an essential function in the regulation of seed germination and plant growth and development [17]. Ion uptake and transport, stomatal conductance, the photosynthetic rate, and transpiration [18,19] could also be affected by SA application. Exogenous SA application to stressed plants, either through seed soaking, adding to the nutrient solution, irrigation, or spraying, has been reported to augment stress tolerance-mechanisms [20,21] against metals [22], salinity [13], drought [23], and heat stress [24]. A direct physiological impact of SA is the modification of antioxidant enzyme actions to increase plant responses to these stresses [25]. Moreover, SA increases plant growth under salt stress, which could be attributed to considerable enhancement in the net photosynthetic rate [26]. El-khodary [27] observed a considerable enhancement in growth characteristics, pigment contents, photosynthetic rate, and carbohydrate content in maize sprayed with SA. Additionally, SA has been shown to ameliorate salt stress in seedlings of cucumbers [28] and cowpea plants [14]. 

With the purpose of improving whole-plant performance under amplified severity of salt stress, more information is required about the contribution of SA to salt stress tolerance in plants at the physiological level. Among the various plants tested, fenugreek (*Trigonella foenum gracium* L.) is an annual leguminous herb regarded as a multi-purpose crop, that is extensively cultivated in most regions of the world for its medicinal value [29]. The use of different parts of the fenugreek plant as flavoring agents, stabilizers, adhesives, or for cosmetics, papers, and paints has also been documented [30]. It is considered a salt-sensitive plant species which is harmfully affected by salt stress in terms of physiology and growth [31,32]. Therefore, this experiment aimed to determine whether exogenous SA application could convey salt tolerance to fenugreek plants at the physiological level, based on various biochemical parameters, osmolytes, and antioxidative enzymes.

## 2. Results

### 2.1. Chlorophyll Content Index 

The results in Table 1 reveal that CCI decreased with increasing salt concentration; however, the foliar application of SA had prominent effects, and an increase in the CCI of fenugreek leaves was observed. 

### 2.2. Total Soluble Carbohydrates

Table 1 shows carbohydrates accumulation in NaCl-treated fenugreek plants, compared to the control. Further accumulation of carbohydrates was detected in NaCl-treated and non-treated plants with foliar application of SA, but in salinized plants (75 and 150 mM NaCl) this accumulation was significantly higher relative to the control. Indeed, with SA application, a significant increase by 8.4% and 7.8% in carbohydrate content was observed in plants grown under 75 and 150 mM NaCl, respectively, as compared with salinized plants only. 

### 2.3. Total Free Amino Acid Content

**Figure 1:**
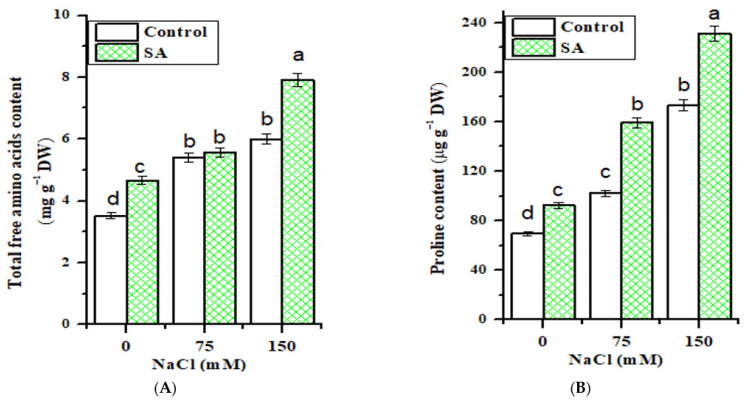
Effect of NaCl concentrations and salicylic (SA) application on (**A**) total free amino acid content and (**B**) proline content of fenugreek plant leaves. Values represent the mean of three replicates. Different letters (a, b, c and d) indicate statistical differences at 5% probability according to Tukey’s test. Error bars are standard errors of the mean. DW—dry weight.

Figure 1A reveals a significant (*p* ≤ 0.05) increase in the total free amino acid content due to NaCl treatment and SA application, compared to control. Although salt stress increased the free amino acid content in fenugreek leaves, this increase was more conspicuous with SA application, realizing a maximum (7.920 mg g^−1^ DW) increase at 150 mM NaCl treatment over salinized plants only (5.999 mg g^−1^ DW).

### 2.4. Proline Content

The results in Figure 1B indicate that the proline content increased in response to NaCl stress, and the proline level was augmented with increasing salt concentration compared to control. Furthermore, with SA application, a significant increase (*p* ≤ 0.05) in proline content in salt-stressed fenugreek plants at the levels of 75 and 150 mM NaCl concentration was detected, when compared with their respective salinized plants only.

### 2.5. Total Phenolics and Flavonoids

**Figure 2:**
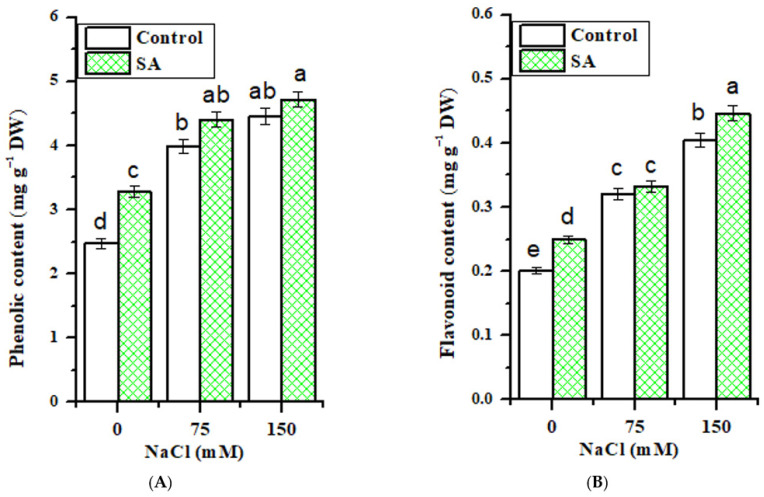
Effect of NaCl concentrations and salicylic (SA) application on (**A**) phenolic content and (**B**) flavonoid content of fenugreek plant leaves. Values represent the mean of three replicates. Different letters (a, b, c, d and e) indicate statistical differences at 5% probability according to Tukey’s test. Error bars are standard errors of the mean. DW—dry weight.

The data of the total phenolic and flavonoid contents in the fenugreek leaves are shown in Figure 2. With increasing salt concentration, there was a considerable increase in total phenolic and flavonoid contents in the fenugreek leaves. Further increases in their contents were observed with SA application, in either control or salt-treated plants. For instance, at 150 mM NaCl, the total phenolic content was about 2-fold (4.716 mg g^−1^ DW) higher than that of the control (2.473 mg g^−1^ DW) due to SA spraying (Figure 2A). In plants challenged by 75 and 150 mM NaCl, the increases in the flavonoid content were 59.3% and 101%; these percentages were boosted by 65.3% and 122%, respectively, with SA application versus control in untreated plants only (Figure 2B). 

### 2.6. Lipid Peroxidation 

**Figure 3:**
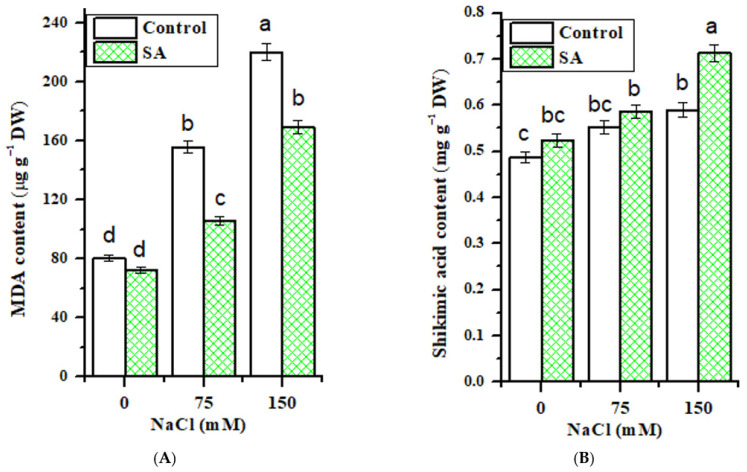
Effect of NaCl concentrations and salicylic (SA) application on (**A**) malondialdehyde (MDA) content and (**B**) shikimic acid content of fenugreek plant leaves. Values represent the mean of three replicates. Different letters (a, b and c) indicate statistical differences at 5% probability according to Tukey’s test. Error bars are standard errors of the mean. DW; dry weight.

Results of the present study revealed significant changes in MDA content in salt-stressed fenugreek leaves, influenced by SA application (Figure 3A). It was noted that with SA application, MDA content was significantly lower than in non-SA applied plants. On the other hand, there was a significant (*p* ≤ 0.05) increase in MDA content with increasing salt concentrations. In non-SA applied plants, an increase by 93.6% and 173.9% in MDA content was observed at 75 and 150 mM NaCl, respectively. Conversely, a lower MDA content was detected with SA spraying (by 32.2% and 23.1%) at 75 and 150 mM, respectively, versus salinized plants only. 

### 2.7. Shikimic Acid Content

Among the tested parameters observed under stress conditions, the highest fluctuations in values of shikimic acid content were observed as a response to salt treatment. The increase in shikimic acid content ranged from 0.553 to 0.589 mg g^−1^ DW at 75 and 150 mM NaCl, compared to control (0.487 mg g^−1^ DW, Figure 3B). Of particular note, a further increase in its content was observed with foliar application of 0.5 mM SA, either in control or salt-treated plants. However, in salt-treated plants, this enhancement was significantly greater relative to the control realizing 0.586 to 0.713 mg g^−1^ DW at 75 and 150 mM NaCl. Further, in terms of two-way ANOVA results (Table 2) for shikimic acid content in the leaves of fenugreek plants, significant changes were observed between salt, SA treatment, and their interactions.

### 2.8. Antioxidative Enzymes 

**Figure 4:**
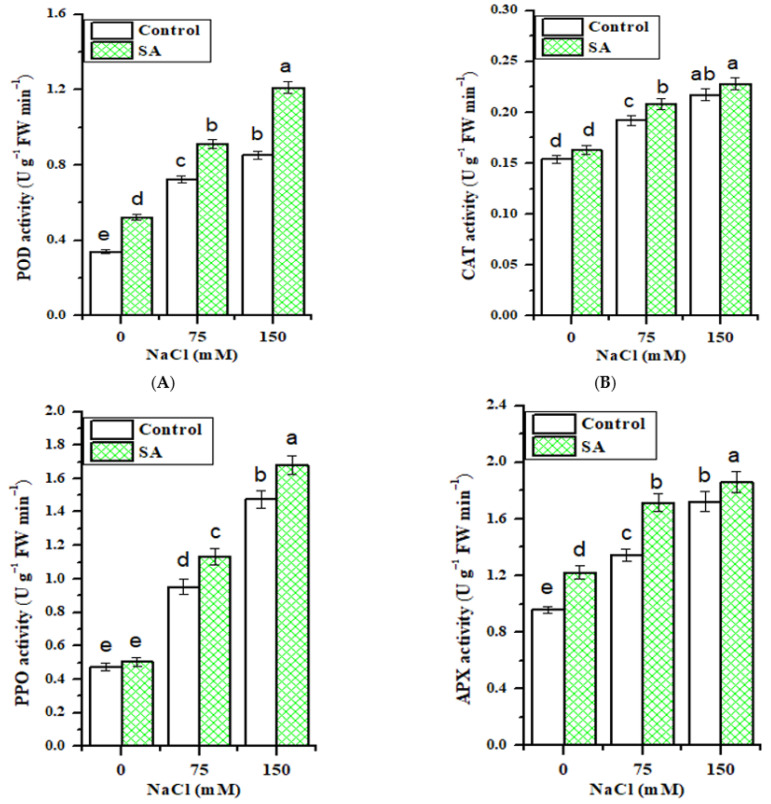
Effect of NaCl concentrations and salicylic (SA) application on antioxidant enzymes activities; (**A**) peroxidase (POD), (**B**) catalase (CAT), (**C**) polyphenol oxidase (PPO) and (**D**) ascorbate peroxidase (APX), of fenugreek plants. Values represent the mean of three replicates. Different letters (a, b, c, d and e) indicate statistical differences at 5% probability according to Tukey’s test. Error bars are standard errors of the mean. DW; dry weight.

Salt stress significantly induced an increase in the enzymatic antioxidant profiles of fenugreek leaves, including POD, CAT, PPO, and APX activities (Figure 4). Moreover, the application of SA significantly (*p* ≤ 0.05) improved their activities (Figure 4A–D), either in control or NaCl-stressed fenugreek plant leaves. Under normal conditions, SA application significantly increased APX and POD activities (by 27.6% and 53.5%, respectively) in comparison with control. At 150 mM NaCl, SA application significantly augmented the activities of POD, APX, and PPO in fenugreek leaves by 42.1%, 8.1% and 14.1%, respectively, as compared to NaCl-treated plants alone. Most interestingly, based on two-way ANOVA results (Table 2), significant differences were established among salt, SA treatment, and their interactions in the antioxidant enzymes of fenugreek plant leaves.

## 3. Discussion

Chlorophyll content is widely used as an indicator of abiotic tolerance in plants, as changes in chlorophyll content are linked to visual symptoms of plant illness and photosynthetic efficiency [33]. A decline in chlorophyll content in salt-applied fenugreek plants might be ascribed to the possible oxidation of chlorophyll and other chloroplast pigments, coupled with the instability of the pigment-protein complex under salt stress [34]. Similar results were obtained in cowpea plants by [14] and [35] under salt stress conditions. Reduction in the chlorophyll content can be reversed back to a similar level as the control by the foliar application of SA, which might account for the induction of Rubisco activity and synthesis of more carbohydrates in treated plants [24]. SA application may also inhibit the production and accumulation of ROS in plant tissues, which negatively affects the photosynthetic unit. 

The increase in carbohydrate content in fenugreek plants grown in soil treated with 75 and 150 mM NaCl could be related to the major role of these carbohydrates in stress alleviation, involving osmoprotectants, carbon storage, and signal molecules. It was reported that carbohydrates such as sugars (e.g., glucose, fructose, fructans, and trehalose) and starch are involved in the response to several stresses, and act as nutrient and metabolite signaling molecules [36,37]. Additionally, the high carbohydrate accumulation helps to prevent oxidative losses by scavenging ROS and maintaining protein structure during salt stress. Moreover, the marked increase in carbohydrate levels in fenugreek leaves following SA application corroborated the findings of [27,38,39]. SA application deranges the enzymatic system of polysaccharide hydrolysis, which stimulates the hydrolysis of insoluble sugars and creates an osmotic source that is important to osmotic regulation. Interestingly, it can be concluded that SA application ameliorates salinity stress by increasing photosynthetic carbohydrates.

Amino acids are essential molecules that shuttle organic nitrogen through the plant, and play several critical roles in plants, from providing the building blocks of proteins to their central role in cellular and plant physiology [40,41]. The increase in amino acid content in fenugreek plants as a result of salt stress and SA application may be attributed to the changes produced by stress promoting the synthesis of amino acids, inhibiting amino acid degradation, and inhibiting protein synthesis and protein degradation [42]. Moreover, amino acids are involved in glutathione and phytochelatin synthesis, which play roles in metal binding and synthesis of polyamines, acting as signaling molecules and antioxidants [43]. Therefore, it could be hypothesized that amino acids contribute to maintaining the cellular osmotic potential, and may have an important role in tolerance to salt stress.

Generally, proline accumulation is an important physiological index for plant response to salt stress and is supposed to correlate with adaptation to salinity [44]. The obtained result of increasing proline content in fenugreek leaves supports earlier reports by [45], in which salinity displayed a conspicuous increase in compatible solutes, such as proline. Hence, the accumulated proline level under salt stress may be due to the activation of proline biosynthesis, which enhances protein turnover [30]. A noteworthy finding from our study is that the use of the plant-growth-hormone (SA) significantly increased the proline content in fenugreek plants under both saline and non-saline conditions, as previously reported by [46] in chickpea plants. Plants require a greater accumulation of energy-rich compounds under a saline soil environment. From these compounds, proline serves as a nitrogen and/or carbon source, or as a building block for peptide and protein synthesis [47]. Moreover, proline acts as a scavenger of free radicals [48], and a cytoplasmic osmolyte that reduces the osmotic potential of the cell and the uptake of toxic ions [49]. 

Similarly, phenolic compounds, a group of secondary metabolites, have different biological activities, and their most important capability is their antioxidant activity in plants [50,51]. Our study indicated the role of SA application in minimizing the harmful effects of salt stress, with evidence of an increase in the protective effects of these compounds against salt stress, supporting plants to maintain ROS levels under detrimental conditions, and facilitating rapid elimination so that metabolism remains stable.

Moreover, the results of a flavonoid increase in fenugreek leaves agreed with earlier studies of [52] on artichoke (*Cynara scolymus* L.) leaves, which may be explained by the finding that enzymes of the flavonoid biosynthesis pathway, such as phenylalanine ammonia-lyase and chalcone synthase, increase under stress [53]. Additionally, flavonoids directly scavenge ROS, including H_2_O_2_, O_2_^−^ and OH^−^ radicals. Furthermore, flavonoids were found to enrich plant tolerance against salinity by regulating K^+^ maintenance and Na^+^ elimination from leaf mesophyll in broad beans and quinoa [54]. 

Lipid peroxidation is the process whereby free radicals steal electrons from the lipids in cell membranes, leading to a free radical chain reaction mechanism that indicates the magnitude of the oxidative stress, resulting in cell damage and the formation of MDA [55,56,57]. The accumulation of MDA in fenugreek leaves subjected to salt stress was ascribed to the production of ROS in cells, which causes peroxidation in membranous lipids and the formation of MDA. The reduction in its content in fenugreek plants following SA application could be attributed to the fact that SA supports plants to produce antioxidative compounds and/or enzymes that diminish ROS before peroxidation [14].

Interestingly, the increase in shikimic acid in fenugreek leaves subjected to salt stress and sprayed with SA was in agreement with the previously observed results of [58,59, 60] for maize, Eucalyptus and carrot, respectively, under different stress conditions. It was proven that the accumulation of shikimic acid depends on the rate of ongoing metabolic processes [61], which leads to the synthesis of plant phenolic compounds via the shikimic acid pathway. This pathway simply converts simple carbohydrate precursors derived from glycolysis and the pentose phosphate pathway into aromatic amino acids, including the SA precursor phenylalanine [62], which could be correlated with the pivotal effect that SA exerts during the plant response to salinity.

The increase in antioxidant enzyme activity in fenugreek plant leaves under salt stress confirms earlier reports [38]. Salinity led to a prominent increase in antioxidant enzymes, which may be due to the reality that ROS are produced in response to different environmental stresses, including salt stress [63,64]. 

Of particular note, a significant (*p* ≤ 0.05) increase in antioxidant enzyme activity following SA application could be the sign of buildup of a protective means to lessen the oxidative damage induced by salt stress. A similar observation was recorded by [65] on soybean plants under the combination of salt stress and SA application, and by [66] under Cd stress. The augmentation of oxidative stress caused by salinity may be overwhelmed by this enzymatic system, and this explains our previously mentioned result of a decrease in MDA content in SA applied fenugreek plants. 

## 4. Materials and Methods

### 4.1. Soil, Seed, and Growth Conditions

The experimental soil was collected from the top layer (0–20 cm) from Sharkia Governate, Egypt. Physicochemical attributes and nutritional status of the experimental soil are recorded in Table 3. Fenugreek seeds (*Trigonella foenum-graecum* L.) (var. Giza 30; procured from Agricultural Research Centre, Giza, Egypt) were surface disinfected with 5% sodium hypochlorite for 10 min, then subsequently washed with distilled water and sown in plastic pots (25 cm diam.) filled with 2 kg of the collected soil. The experiments were kept under controlled conditions (10 h light/14 h dark cycle at 20/15 °C (day/night), moistened repeatedly with water, and thinned to five germinated seeds per pot after 10 days of germination. 

### 4.2. Salt and Salicylic Acid Application

Salt solutions were applied after one month from sowing at the levels of 0, 75 and 150 mM NaCl, then SA was applied after finishing the salt addition by spraying the fenugreek plant leaves with 0.5 mM SA solution, versus no SA (control sprayed with distilled water) in the early morning for seven successive days. Hence, there were six treatments (2 × 3), and each treatment was replicated three times (2 × 3 × 3). The plant samples from all treatments were collected after 4 weeks from SA application and kept in labeled bags for different analysis.

### 4.3. Physiological Evaluation 

#### 4.3.1. Estimation of Chlorophyll

The chlorophyll content index (CCI) in fenugreek leaves was estimated by the CCM-200 plus Chlorophyll Content Meter (Opti-Sciences, Inc., Hudson, NH, USA). CCI results are the mean of the data recorded at different positions along the length of the leaf. 

#### 4.3.2. Estimation of Carbohydrate Content

Carbohydrates were estimated by the phenol sulphuric acid technique, as confirmed by [67]. A known weight of dried fenugreek leaves was extracted with 2.5 N HCl. The absorbance was read at 490 nm. 

#### 4.3.3. Determination of Total Free Amino Acids and Proline Content

The total free amino acid content of fenugreek leaf tissue was assessed quantitatively in an alcoholic extract by the method of [68]. Ninhydrin solution, citrate buffer, glycerol (55%), and 0.5 mL of sample solution were mixed subsequently, and the volume was brought up to 5 mL by distilled water. After heating in a boiling water bath for half an hour, the mixture was then cooled under tap water and gently shaken. The absorbance was read at 570 nm. 

The proline content of fresh leaves was extracted in 3% aqueous sulphosalicylic acid, and the homogenate was filtered. Two mL of glacial acetic acid and 2 mL of acid ninhydrin were added to 2 mL of the filtrate in a glass test tube and heated in a boiling water bath for 1 h, and then were placed in an ice bath. Four mL of toluene was added to the reaction mixture and stirred well for 20–30 s. The optical density of the produced color was measured at 520 nm [69]. 

#### 4.3.4. Determination of Total Phenolic and Flavonoid Contents

Total phenolic content in fenugreek leaves was determined [70] after 95% ethanol extraction; then, 1 mL from the extract was mixed with 1 mL of Folin reagent and 1 mL of Na2CO3 (20%). The absorbance was computed at 650 nm. 

Total flavonoid content in fenugreek leaves was assayed according to [71], using the aluminum chloride colorimetric method. A 100 μL aliquot of the alcoholic extract was added to 4 mL of distilled water. At zero time, 0.3 mL 5% sodium nitrite was added. After 5 min, 0.3 mL of 10% aluminum chloride was added. After 6 min, 2 mL of 1 M sodium hydroxide was added to the mixture. Absorbance was measured at 510 nm.

#### 4.3.5. Determination of Malondialdehyde (MDA) Content 

Lipid peroxidation rates in fresh fenugreek leaves were determined by measuring the MDA content according to the method of [72], using thiobarbituric acid. Absorbance was determined at 450, 532, and 600 nm, respectively, using a spectrophotometer, and its content was estimated based on the following formula:

MDA (μm/L) = 6.45(A_532_ − A_600_) − 0.56 A_450_

#### 4.3.6. Shikimic Acid Content

Fresh fenugreek leaves were homogenized in 0.25 M HCl (1 mL/100 mg biomass) [73] and centrifuged at 3000 rpm for 30 min. Then, 0.5 mL of 1% periodic acid was added to 50 µL of supernatant and left for 3 h at room temperature. After that, 0.5 mL of 1 M sodium hydroxide and 0.3 mL of 0.1 M glycine were added, and the samples were centrifuged again. The absorbance of the samples was read at 380 nm.

#### 4.3.7. Estimation of the Antioxidant Enzymes 

A known shoot fresh weight of fenugreek was ground in 0.05 M phosphate buffer (pH 7.0) containing 1 mM EDTA (Ethylene Diamine Tetra Acetic Acid), and centrifuged at 10,000 rpm for 10 min. The supernatant was finalized to a total known volume and used as an enzyme source. An assay of catalase activity (CAT) was undertaken in line with the method of [74]. Peroxidase activity (POD) was estimated based on information from [75], wherein 5 mL of the assay mixture, comprising 300 µM phosphate buffer (pH 6.8), 50 µM catechol, 50 µM H_2_O_2_ and 1 mL of enzyme extract, was prepared. The optical density was measured at 430 nm.

An assay of polyphenol oxidase activity (PPO) was performed according to [76], wherein 5 mL of assay mixture comprising 125 µM of phosphate buffer (pH 6.8), 100 µM of pyrogallol, and 1 mL of enzyme extract were prepared. The optical density of the produced color was measured at 430 nm. Ascorbate peroxidase (APX) activity was prepared and assayed according to [77]. 

### 4.4. Statistical Analysis

Origin 2017 was used for data processing and forming figures. The data were subjected to statistical studies using a two-way analysis of variance (ANOVA), using the Statistical Package for the Social Science (SPSS) version 15.0. The data are presented as the means ± standard errors of at least three replicates per treatment (*n* = 3), and the different letters indicate the statistical differences at *p* < 0.05 using the post hoc test (Tukey’s test).

## 5. Conclusions

It may be concluded from our results that NaCl had damaging effects on the chlorophyll content and biochemical parameters of fenugreek plants. SA application alleviates the devastating effects generated by salt stress, and this was evidenced by the increase in CCI, carbohydrates, phenolics, flavonoids, and total free amino acids. Increases in shikimic acid, proline, and antioxidant enzyme activity, and a lower MDA content, were also detected with SA application. Thus, it may be concluded that exogenous SA application might act as a powerful tool in enhancing physiological parameters and protecting plants from abiotic stresses, such as salt stress.

## Figures and Tables

**Table 1:** Effect of NaCl concentrations and salicylic (SA) application on the chlorophyll content index (CCI) and carbohydrate content of fenugreek plant leaves.

NaCl (mM)	SA (mM)	CCI	% C	Carbohydrates (mg g^−1^ DW)	% C
0	0	22.03 ± 0.91 ab	11.3	79.2 ± 4.5 c	4.3
	0.5	24.51 ± 3.08 a		82.6 ± 4.6 bc	
75	0	17.87 ± 1.11 b	35.3 ↑	91 ± 5.1 bc	8.4 ↑
	0.5	24.17 ± 4.61 a		98.6 ± 5.5 ab	
150	0	13.27 ± 3.56 c	32.1 ↑	110.4 ± 6.1 a	7.8 ↑
	0.5	17.53 ± 3.65 b		119 ± 7.3 a	

Data shown in the table represent the mean ± standard error, followed by a small letter; similar letters indicate that means were not different significantly at 5%, probability based on Tukey’s test. %C means SA-treated plants expressed as % of their respective non-treated controls. DW— dry weight, ↑ means increase.

**Table 2:** Analysis of variance of the effect of salt, salicylic (SA), and their interactions on some physiological parameters in fenugreek leaves, based on a two-way ANOVA analysis.

Parameters	Salt	SA	Salt × SA
Chlorophyll content index	*	*	*
Carbohydrates	*	ns	ns
Lipid peroxidation	*	*	*
Phenolics	*	*	ns
Flavonoids	*	*	ns
Shikimic acid	*	*	*
Total free amino acids	*	*	*
Proline	*	*	*
POD	*	*	ns
CAT	*	*	ns
APX	*	*	*
PPO	*	*	*

ns (non-significant) and * (significant at the 5% probability level).

**Table 3:** Physicochemical properties and nutritional status of the experimental soil before planting.

Property	Sand (%)	Silt (%)	Clay (%)	Soil Texture	Saturation Percent	Electric Conductivity	pH	CaCO_3_ (%)	Mineral Content				Organic Matter
									(mg/kg Soil)			(%)	
									K	Mg	Ca	Total P	(%)
Value	13.9	27.4	58.7	clay	69	3.4	8.2	4.9	0.37	6.3	8.4	0.69	1.2

## Data Availability

No new data were created or analyzed in this study. Data sharing is not applicable to this article.
